# Age-related functional changes of total thyroid hormones and glycosaminoglycans in growing calves

**DOI:** 10.14202/vetworld.2020.681-686

**Published:** 2020-04-14

**Authors:** Pietro Medica, Cristina Cravana, Alida Maria Ferlazzo, Esterina Fazio

**Affiliations:** 1 Department of Veterinary Sciences, Unit of Veterinary Physiology, Polo Universitario Annunziata, Messina University, 98168 Messina, Italy; 2 Department of Veterinary Sciences, Unit of Veterinary Biochemistry, Polo Universitario Annunziata, Messina University, 98168 Messina, Italy

**Keywords:** calves, glycosaminoglycans, growth, thyroxine, triiodothyronine

## Abstract

**Background and Aim:**

During the physiological growing, thyroid and proteoglycan glycosaminoglycan (GAG) changes dynamically occur, according to genetic and non-genetic factors. The purpose of this research was to compare the effects of early postnatal development (10 days) until 210 days of life on the triiodothyronine (T_3_), thyroxine (T_4_), the relative T_4_:T_3_ ratio, and GAGs profile, and to define the different reference intervals of the calf’s development through the various growing phases.

**Materials and Methods:**

The effect of growing on total thyroid hormones and GAG profiles was studied from 10 days to 210days of age in 64 clinically healthy Brown calves, 30males and 34females. Blood samples were collected at 10, 20, 30, 60, 90, 120, 150, 180, and 210days of age.

**Results:**

The results showed a significant effect of a calf’s growth on T_3_, T_4_, and GAG values (p<0.0001). Significant correlations between T_3_ and T_4_ were observed. Compared to the previous time point, T_3_ showed a significant decrease at 20days and at 60days (p<0.01), while a significant increase was observed at 90days and 210days (p<0.05); T_4_ showed a significant decrease at 20days (p<0.01), while significant increases were observed at both 180days and 210days (p<0.05); GAGs showed a significant increase at 120days and 210days (p<0.05). Positive and significant correlations between BW and GAGs in both males (p<0.0057) and females (p<0.0059) were observed.

**Conclusion:**

It can be concluded that the highest T_3_ and T_4_ concentrations have been associated with the early growing process (10days), with an increasing trend also at 210days, it is possible to hypothesize a probable metabolic effect of thyroid function in anabolic and/or catabolic directions during the calves’ development. Likewise, it can be reasonably inferred that the highest plasma GAGs at 210days may be due to their metabolic role during the development of growing calves. Taken together, these findings suggest the potential and relative contribution made by thyroid and GAGs effects on the dynamics of growing calves.

## Introduction

The hypothalamic-pituitary-thyroid (HPT) axis plays a consistent role in the growth and development of fetal and neonatal calves [1,2], according to metabolic and non-metabolic mechanisms [3-5]. Indeed, thyroid hormones play a crucial role in successful implantation and during the early stages of embryo development [6,7], suggesting a local action of thyroid hormones and thyroid-stimulating hormone (TSH) on both the endometrium and the embryo [8,9]. Several reports indicated that the fetal number also affected the circulating thyroid hormones during pregnancy of ewes [10-12].

Fetal thyroid function is under a strong maternal thyroid axis’s influence, on the basis of placental permeability for iodine, improving fetal thyroid hormone synthesis [13,14]. Nevertheless, there is no evidence about thyroid hormones transfer through the ruminants’ placenta [2]. During the physiological development, thyroid changes dynamically occur, according to genetic and non-genetic factors, nutritional intake [15], environmental stimuli [16], and deiodinases’ expression [2]. High fetal thyroid hormones’ concentration after birth in 1day old neonatal calves were observed, and a significant decrease along the next several days was also recorded, providing sufficient amounts of thyroid hormones for early postnatal development [17]. An interplay between fetal thyroid function and skeletal muscle development, in both sheep [4,18] and cattle [19], and the initiation of neonatal thermogenesis [20], was reported.

Glycosaminoglycans (GAGs) are heteropolysaccharides, widely distributed in mammals’ tissues, which display varied stereochemistry, chain lengths, and patterns of sulfatation. GAG-protein interactions participate in neuronal development, angiogenesis, and other functions such as immune responses [21]. Recently, the implications for proteoglycan signaling, and the identification of novel binding sites in receptor protein-tyrosine phosphatase in modulating neural development and regeneration, were also suggested [22]. Fetal calf serum supported high levels of [3H] glucosamine incorporation into hyaluronic acid, in a dose-dependent manner, while newborn calves and calf sera supported much lower levels of incorporation [23]. It is also worth noting that proteoglycans in endochondral ossification were observed and that they appear to persist unaltered in the calcified cartilage core of the trabeculae, until the primary spongiosa is replaced by the secondary spongiosa [24]. There are age and site related differences in the extent of proteoglycans isolated aggregates in cephalic, epiphyseal, and articular cartilages, in growing sheep [25] and deer (*Cervus Nippon*) [26].

The hypothesis of the work was the supposed existence of functional differences in the same individuals during the 1^st^210days of life, reflecting the metabolic shift from the onset of growth and the dynamic development of neonatal calves. The primary objective of the present research was to compare the effect of early postnatal development (10days) until 210days of life on the total thyroid hormones, the relative T_4_:T_3_ ratios, and GAGs profile. The secondary one was to establish whether changes in the ranges of circulating compounds were suitable to define different reference intervals of the calf throughout the growing phases.

The current study therefore underlines the importance of these parameters, with consistent metabolic effects, throughout the growing phases, coupled with information on their comparative involvement in interpreting physiological or clinical conditions.

## Materials and Methods

### Ethical approval

All methods and procedures used in this study were in compliance with the guidelines of Italian law (D.L. 04/3/2014 n. 26) and EU directive (2010/63/EU) on the protection of animals used for scientific purposes.

### Animals

Sixty-four clinically healthy Brown calves, 30males and 34females, born in early November and kept out with dams until weaning, were investigated during the 1^st^210days of age, from 10 to 210days. The calves were fed on their dam’s milk until weaning and all calves were weaned at 6months.

During the experimental period, individual live body weights (BW) were recorded monthly using a large animal scale.

### Samples collection

Calves were sampled on a farm located North Sicily (38° 1’ 51’’ 20N latitude, 15°7’ 57’’ 72E, longitude) between March and September, 2018. Blood samples were taken from the jugular vein at 10, 20, 30, 60, 90, 120, 150, 180, and 210days of life, respectively. All samples were taken between 07:00 and 09:00 a.m. to minimize the effect of circadian rhythm on hormonal measurements. During blood collection, the animals received minimum handling, and minimal physical restriction was involved to reduce handling stress. Blood samples were collected in quiet conditions by the same veterinarian, by jugular venipuncture and drawn into a plain vacutainer tube for serum (Venoject, Terumo^®^; Belgium). The blood samples were centrifuged for 15min at 1500× *g*, and serum was separated. The serum samples were stored frozen in polystyrene tubes at -20°C and assayed for thyroxine and triiodothyronine. For plasma preparation, blood was collected into tubes containing EDTA and then separated by centrifugation at 1500× *g*. The samples were either immediately processed for GAGs separation, or transferred to plastic vials and stored at -20°C until analysis.

### Sample analysis

Thyroid hormone assays were analyzed in duplicate using a commercially available immunoenzymatic kit and carried out according to the manufacturer’s instructions (SEAC-RADIM; Pomezia, Rome). Limits of detection were 0.24 nmol/L for T_3_ and 5.79 nmol/L for T_4_. Intra-assay and inter-assay coefficients of variation were 7.3% and 11.4% for T_3_, 2.3% and 5.7% for T_4_, respectively, on the basis of measurements, in three different samples. The commercial kits were validated for total iodothyronines by establishing that dilutions of ovine serum resulted in curves identical to those obtained with the human standards supplied with the assay kits.

Isolation of GAGs from plasma used reagents of analytical grade (Merck, Darmstad, Germany; Fluka, Buchs, Switzerland; Sigma, St. Louis, MO, U.S.A.). The ion-exchanger ecteola-cellulose was from Fluka and standard sugars from Sigma. The GAG isolation from plasma preparations was performed as described in detail elsewhere [27]. Aknown amount (1-5ml) of plasma, diluted to double volume with water, was held in alkaline conditions (0.05 M NaOH) at 40°C for 16h to cleave covalent O-linkages between protein and carbohydrate and to release GAG chains from proteoglycans or peptidoglycans [28]. The sample, cooled to room temperature, was neutralized to pH6-6.5 by adding 1M HCl solution. GAG chains were then isolated by filtering the neutralized samples through columns (0.7 × 4cm columns were used for 2ml samples) of the weak anion exchanger Ecteola-cellulose (Fluka), in chloride form. The resin was washed with 50ml of 0.15 M NaCl solution and then GAGs were eluted by 4ml of 2M NaCl solution and quantified in terms of hexuronic acid.

### Statistical analysis

Data are presented as mean ± standard deviation (S.D.). To analyze differences for previous time points, one-way analysis of variance for repeated measures (one-way RM ANOVA) was applied. When the F value was significant, differences between individual means were assessed with a *post hoc* test (Bonferroni). Significant differences between males and females were established using Student’s unpaired t-test. The level of significance was set at p<0.05. All calculations were performed using the GraphPad Prism version7.0 for Windows (GraphPad Software Inc., San Diego, CA, USA). The correlations between BW measurements, age, thyroid hormones, and GAGs were calculated using the Pearson’s linear regression, r. The correlations between age, thyroid hormones, and GAGs were also evaluated.

## Results

### Growing effect

**Figure-1:**
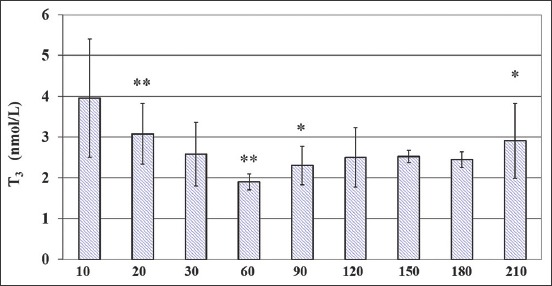
Circulating total triiodothyronine (T3) concentrations (M ± SD) in growing calves over a period of 210days. *Indicates significant (*p<0.05; **p<0.01) differences in average hormone concentrations versus previous time point.

**Figure-2:**
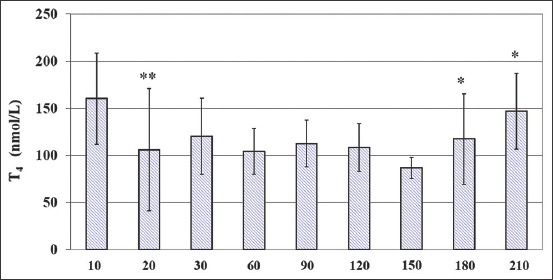
Circulating total thyroxine (T_4_) concentrations (M ± SD) in growing calves over a period of 210days. *indicates significant (*p < 0.05; **p < 0.01) differences in average hormone concentrations vs previous time point.

**Figure-3:**
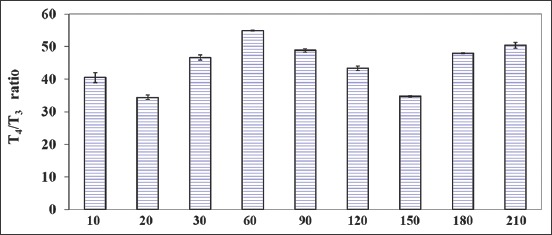
T_4_/T_3_ ratio in growing calves over a period of 210days.

**Figure-4:**
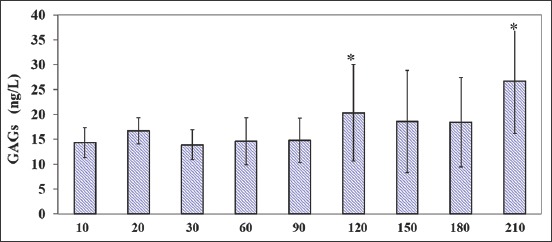
Circulating glycosaminoglycan concentrations (M ± SD) in growing calves over a period of 210days. *indicates significant (*p<0.05) differences in average hormone concentrations versus previous time point.

The growth in BW, total iodothyronine, T_4_/T_3_ ratio, and GAG concentrations in calves from 10 to 210days of age is, respectively, shown in Table-1 and Figures-1-4. The effect of growth was observed for T_3_ (F=1.25; p<0.0001), T_4_ (F=1.46; p<0.0001), and GAG (F=17.24; p<0.0001) values.

**Table-1:** Growth in body weight (M±SD) in growing calves from 10 to 210 days of age.

Age (days)	BW (kg)	

	Males (30)	Females (34)
10	10.09±2.23	8.50±1.14
20	8.02±1.44	7.53±1.17
30	14.22±3.53	13.64±2.90
60	35.44±3.62	34.33±3.39
90	43.89±1.88	44.67±0.97
120	17.80±1.38	17.13±1.45
150	40.81±2.38	38.93±2.45
180	17.41±1.62	18.33±1.39
210	128.19±35.88	158.17±33.47

Serum T_3_ concentrations averaged, respectively, 3.96 and 1.90 nmol/L at 10 and 60days, showing a two-phase trend, with a decrease from 20days to 60days and a trend to increase from 90days to 120days. Specifically, compared to the previous time point, T_3_ showed lower concentrations at 20days (p<0.01) and 60days (p<0.01) of age, and higher concentrations at 90days and 210days (p<0.05).

Serum T_4_ concentrations averaged, respectively, 160.23 and 86.74 nmol/L at 10days and at 120days, showing a three-phase trend, with a decrease at 20days, a plateau from 30days to 150days, and an increase at both 180days and 210days. Specifically, compared to the previous time point, T_4_ showed lower concentrations at 20days (p<0.01) and significant increases at both 180days and 210days (p<0.05).

Significant correlations between T_3_ and T_4_ along 210days of age (r=0.71; p=0.029) were observed.

Plasma GAGs concentrations averaged, respectively, 30.60 and 10.21ng/L at 210 and 60days, showing a two-phase trend, with the lower concentrations from 10days to 90days, in comparison with values observed from 120days to 210days. Specifically, compared to the previous time point, GAGs showed a significant increase at 120days and 210days (p<0.05).

The average values of T_4_:T_3_ ratio in growing calves were 54.86:1 and 34.42:1 at 60days and 20days, respectively, showing a variable trend, with a decrease at 20days, a plateau from 30days to 120days, a decrease at 150days and an increase at both 180days and 210days.

### Gender effect

No gender effects (p>0.05) were shown for T_3_, T_4_, and GAG concentrations. Males and females showed the typical trend of total iodothyronines and GAGs observed in total calves. No significant differences were observed between males and females for total thyroid hormones and GAG concentrations.

In addition, there were positive correlations between BW and GAGs in both males (r=0.83; p<0.0057) and females (r=0.83; p<0.0059).

## Discussion

Reliable reference values for thyroid hormone concentrations in blood of clinically normal animals have been established by many researches, but limited data are available for GAGs [27]. In fact, many physiological factors that affect thyroid function and GAG metabolism may lead to misinterpretation of average results when values for individual specimen are compared with reference values. Comparisons of total iodothyronine and GAG concentrations with published data for calves under 3months [29] and cattle [27] did not reveal any large discrepancies. However, slight variation might be ascribed to differences in sample collection and processing or laboratory analyses; some differences may be also explained by age, nutritional, managing, or environmental factors.

The obtained data confirm the presence of high concentrations of circulating T_3_ and T_4_ hormones in calves, previously reported in the calves and heifers of *Bos frontalis* [30,31], and during the first 6months of life [32]. Furthermore, the highest concentration of T_3_ observed at 10days in calves and then decreasing with advancing age is in accordance with Garg *et al*. [30] and Lalsangpuii *et al*. [31] showing a probably adaptive mechanism to overcome the stressful period compared to other age periods; it is therefore reasonable that a decreased T_3_ metabolic clearance occurred, due to low capability of its degrading enzymatic system observed in heifer [31] and/or to lower metabolic load [32].

The steady high patterns of T_3_ and T_4_ at the 1^st^10days and also at 210days of age, of growing calves could be associated with a concomitant increased synthesis of T_4_ and with the higher monodeiodination rate of T_4_ to T_3_. Besides, the very high plasma GAG’s values at 210days of life appear to have a consistent and concomitant metabolic role involved in the development and differentiation of young growing calves.

Moreover, serum T_3_ and T_4_ concentrations were relatively stable in growing calves along 120-180days and 30-150days of life, respectively, according to the shift in energy consumption. In fact, circulating T_4_ concentrations were found to be an indicator of energy balance, BW gain, and protein deposition, as reported by Ellenberger *et al*. [33] and Hayden *et al*. [34] in steers.

The key finding in the present study was the time courses of T_4_ and T_3_ changes that were different during the growing period, with a positive correlation between total iodothyronines in the growing calves, confirming this relationship previously described in both buffaloes [3] and goats at different ages [35]. On this basis, T_3_ and T_4_ concentrations appear to be much more susceptible to variations attributable to many exogenous influences, commonly associated with growing calves, than to GAGs trend.

One possibility is that the rise of T_3_ at 90days and 210days and of T_4_ at 180days and 210days, respectively, led to metabolic effects not measured in this research. These data suggest that both T_3_ and T_4_ may contribute to homeostasis during the anabolic processes, and the endocrine changes may, therefore, be the physiological responses to growing itself.

The present data do not completely elucidate the significance of changes in circulating total iodothyronines in calves. However, thyroid changes may be both the cause and the consequence of growth programming.

Changes in plasma GAGs generally followed those for T_3_. However, the changes observed during the 1^st^60days of age were modest and inconsistent.

Total iodothyronines appear to act synergically with GAGs to hasten development especially of the musculoskeletal and nervous system and to improve postnatal adaptation including extremely high thermogenic capacity [36].

The existence of positive correlations between T_3_ and T_4_ concentrations confirms that T_3_ is the most metabolically active iodothyronine [37], especially during normal growth and development, showing a pivotal role in regulation of growth processes and energy metabolism [3,38]. Hence, serum T_4_ concentrations represent a result of the balance between thyroidal secretion and peripheral metabolism that increases according to the growing period [39].

GAGs profile showed a superimposed effect on growing calves, according to different age, playing a crucial key role in physiological development of bone, joint and tooth and signaling events, with a significant relevance to therapeutic options, as showed by the positive correlation between GAGs and body weight.

## Conclusion

It can be concluded that the highest T_3_ and T_4_ concentrations have been associated with the early growing process (10days), with an increasing trend also at 210days, it is possible to hypothesize a probable metabolic effect of thyroid function in anabolic and/or catabolic directions during the calves’ development. Likewise, it can be reasonably inferred that the highest plasma GAGs at 210days may be due to their metabolic role during the development of growing calves. Taken together, these findings suggest the potential and relative contribution made by thyroid and GAGs effects on the dynamics of growing calves.

## Authors’ Contributions

EF and PM conceived the study designed. AMF performed the experiment. PM and CC analyzed the data. EF and PM drafted and revised the manuscript. All authors read and approved the final manuscript.
